# Cancer-associated Fibroblasts induce epithelial-mesenchymal transition *via* the Transglutaminase 2-dependent IL-6/IL6R/STAT3 axis in Hepatocellular Carcinoma

**DOI:** 10.7150/ijbs.45446

**Published:** 2020-07-19

**Authors:** Changchang Jia, Guoying Wang, Tiantian Wang, Binsheng Fu, Yincai Zhang, Lei Huang, Yinan Deng, Guanzhong Chen, Xiaocai Wu, Jianning Chen, Yuhang Pan, Yan Tai, Jinliang Liang, Xuejiao Li, Kunhua Hu, Bo Xie, Sujun Li, Yang Yang, Guihua Chen, Qi Zhang, Wei Liu

**Affiliations:** 1 Cell-gene Therapy Translational Medicine Research Center, The Third Affiliated Hospital of Sun Yat-sen University, Guangzhou, China.; 2 Guangdong Key Laboratory of Liver Disease Research, The Third Affiliated Hospital of Sun Yat-sen University, Guangzhou, China.; 3 Department of Hepatic Surgery and Liver transplantation Center of the Third Affiliated Hospital, Organ Transplantation Institute, Sun Yat-sen University; Organ Transplantation Research Center of Guangdong Province, Guangzhou, China.; 4 Department of medical oncology, The Third Affiliated Hospital of Sun Yat-sen University, Guangzhou, China.; 5 Department of pathology, The Third Affiliated Hospital of Sun Yat-sen University, Guangzhou, China.; 6 Zhongshan School of Medicine, Sun Yat-sen University, Guangzhou, China.; 7 School of Informatics, computing and engineering, Indiana University, Bloomington, IN, USA.

**Keywords:** HCC, CAFs, EMT, TG2, IL-6/IL6R/STAT3 axis

## Abstract

Cancer-associated fibroblasts (CAFs) play crucial roles in enhancing cell survival, proliferation, invasion, and metastasis. We previously showed that hepatocellular carcinoma-derived CAFs (H-CAFs) promoted proliferation of hepatocellular carcinoma (HCC) cells. This study aimed to further explore the role of CAFs in HCC epithelial-mesenchymal transition (EMT) and the underlying mechanism. High CAF density was significantly associated with liver cirrhosis, inferior clinicopathologic characteristics, elevated EMT-associated markers, and poorer survival in human HCC. Within HCC cells, EMT was induced after co-culture with H-CAFs. Secretomic analysis showed that IL-6 and HGF were the key EMT-stimulating cytokines secreted by H-CAFs. Proteomic analysis revealed that TG2 was significantly upregulated in HCC cells with EMT phenotypes. Overexpression of TG2 promoted EMT of HCC cells, and knockdown of TG2 remarkably attenuated the H-CAF-induced EMT. Furthermore, during EMT, TG2 expression was enhanced after HCC cells were stimulated by IL-6, but not HGF. Inhibition of the IL-6/STAT3 signaling decreased TG2 expression. The principal TG2 transcription control element and a potential STAT3 binding site were identified using promoter analysis. Hence, H-CAFs facilitates HCC cells EMT mediated by IL-6, which in turn activates IL-6/IL6R/STAT3 axis to promote TG2 expression.

## Introduction

Hepatocellular carcinoma (HCC) is the fifth most common malignancy and the third leading cause of cancer-related death worldwide 1. Loco-regional invasion and distant metastasis remain the major cause of the high mortality in advanced HCCs, which comprise a significant proportion within all diagnosed cases 2.

Cancer-associated fibroblasts (CAFs) play critical roles in tumor-stroma interaction 3 and is a key modulator of carcinogenesis 4, tumor progression 5, 6, and chemoresistance 7. Knowledge of the interaction between HCC cells and stromal fibroblasts and the underlying mechanism remains limited 8. In our previous study 9, we successfully isolated specific CAFs from primary human HCC tissues (H-CAFs) and demonstrated that H-CAFs suppressed the anti-tumor function of natural killer cells and created a favorable immunosuppressive shield for HCC cells. Moreover, we showed that H-CAFs promoted HCC cell proliferation and prevented HCC cell necrosis and/or apoptosis in extreme environments lacking oxygen and nutrients 10. However, the contribution of CAFs to HCC progression is far from fully understood. Elucidation of the underlying molecular process will be crucial for developing novel targeted therapeutic strategies for HCC.

Epithelial-mesenchymal transition (EMT) is a vital event during the progression of various carcinomas 11, 12. It closely correlates with tumor invasion and metastasis 13, 14. To our knowledge, CAF-induced EMT has rarely been reported in HCC. EMT is often accompanied by increased expression of various transcription factors including transglutaminase 2 (TG2), a multi-functional enzyme which catalyzes the formation of intermolecular isopeptide bonds between glutamine and lysine side-chains 15, 16. The role of TG2 in HCC remains largely unexplored 16.

In this study, we first observed an association of high CAF density with inferior clinicopathologic characteristics, elevated EMT-associated markers, and poorer survival in human HCC, and then showed that co-culture with H-CAFs induced EMT within HCC cells. Secretomic study further showed that interleukin-6 (IL-6) and hepatocyte growth factor (HGF) were the key EMT-stimulating cytokines secreted by H-CAFs, and proteomics analysis revealed that TG2 was significantly upregulated in HCC cells undergoing EMT. The IL-6/signal transducer and activator of transcription 3 (STAT3)/TG2 signaling which played an important role in CAF-induced EMT was then established, and the clinical relevance was confirmed. These findings suggest H-CAFs as a novel vital EMT inducer through secretion of IL-6 and HGF in HCC, and highlight the involvement of TG2 in the IL-6/STAT3-dependent pathway. The key molecules mediating the crosstalk between CAFs and HCC cells and the identified function sites are potential novel prognostic biomarkers and therapeutic targets in HCC.

## Results

### H-CAF density in human HCC tissue associates with tumor progression and patient survival

**Figure 1:**
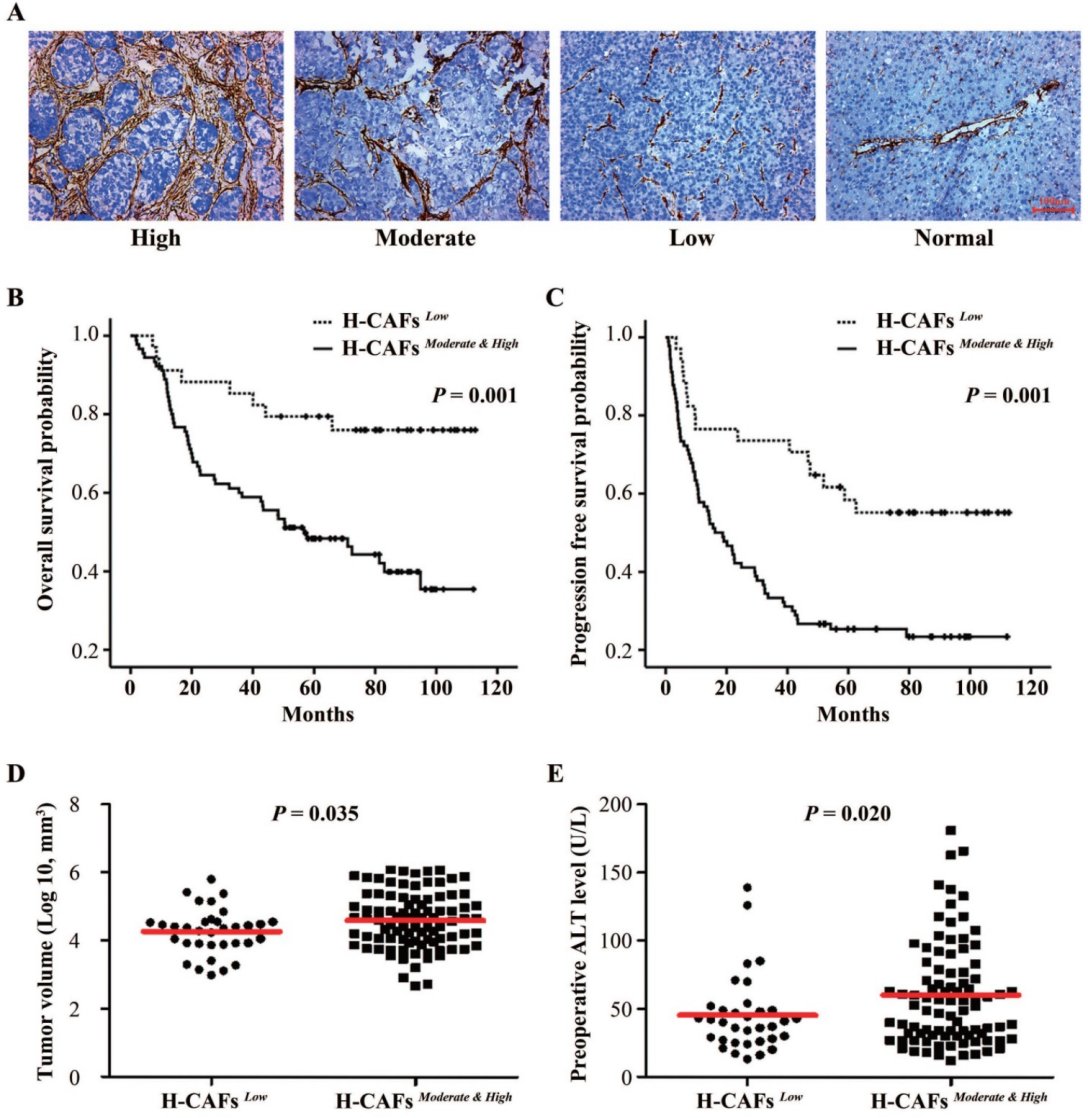
** Correlation of CAF density with prognosis in HCC.** (**A**) CAFs were abundant in the tumor stroma but scarce in normal tissue. We categorized the HCC tissues into three subgroups with high, moderate, and low CAF density (original magnification, 100 ×). (**B and C**) Kaplan-Meier estimation of overall survival and progression-free survival according to the density of H-CAFs in HCC patients. (**D**) Tumor volume and (**E**) preoperative ALT level from HCC patients were also tested.

To explore the role of H-CAFs in HCC progression, we first analyzed the density of H-CAFs in human HCC specimens. H-CAF density was divided into low-, moderate-, and high-density groups based on α-smooth muscle actin (α-SMA) expression (**Figure 1A**). The correlation between H-CAF density and clinicopathologic parameters are shown in **Tables 1 and 2**. An abundance of H-CAFs (moderate-high density) was observed in 72.6% (90/124) of HCC samples. Moderate-high H-CAF density was significantly associated with HBV-infection, liver cirrhosis, and poor-moderate differentiation grade (**Table 1**), and tended to correlate with larger tumor size and more advanced TNM stage. When tumor size was compared as a continuous variable between subgroups with dichotomized H-CAF density, it was higher in the moderate-high density subgroup than in the low density subgroup (**Figure 1D**). Furthermore, the abundance of H-CAFs associated with higher preoperative levels of alanine aminotransferase (ALT) (**Figure 1E**).

Prognostic information was available for all the 124 recruited patients. The 1-, 3-, and 5-year overall survival (OS) rates were 87.1%, 66.9%, and 57.0%, respectively, and the 1-, 3-, and 5-year progression-free survival (PFS) rates were 62.9%, 44.4%, and 30.4%, respectively. Kaplan-Meier analyses demonstrated that a low density of H-CAFs significantly associated with better OS (**Figures 1B**) and PFS (**Figures 1C**). Multivariable analysis revealed that H-CAF density and tumor size were independent prognostic factors for both OS (hazard ratio (HR)=2.318, 95% CI=1.027-5.232) and PFS (HR=2.348, 95% CI=1.259-4.380) (**Table 2**).

### H-CAFs promoted HCC cell invasion and migration by inducing EMT

**Figure 2:**
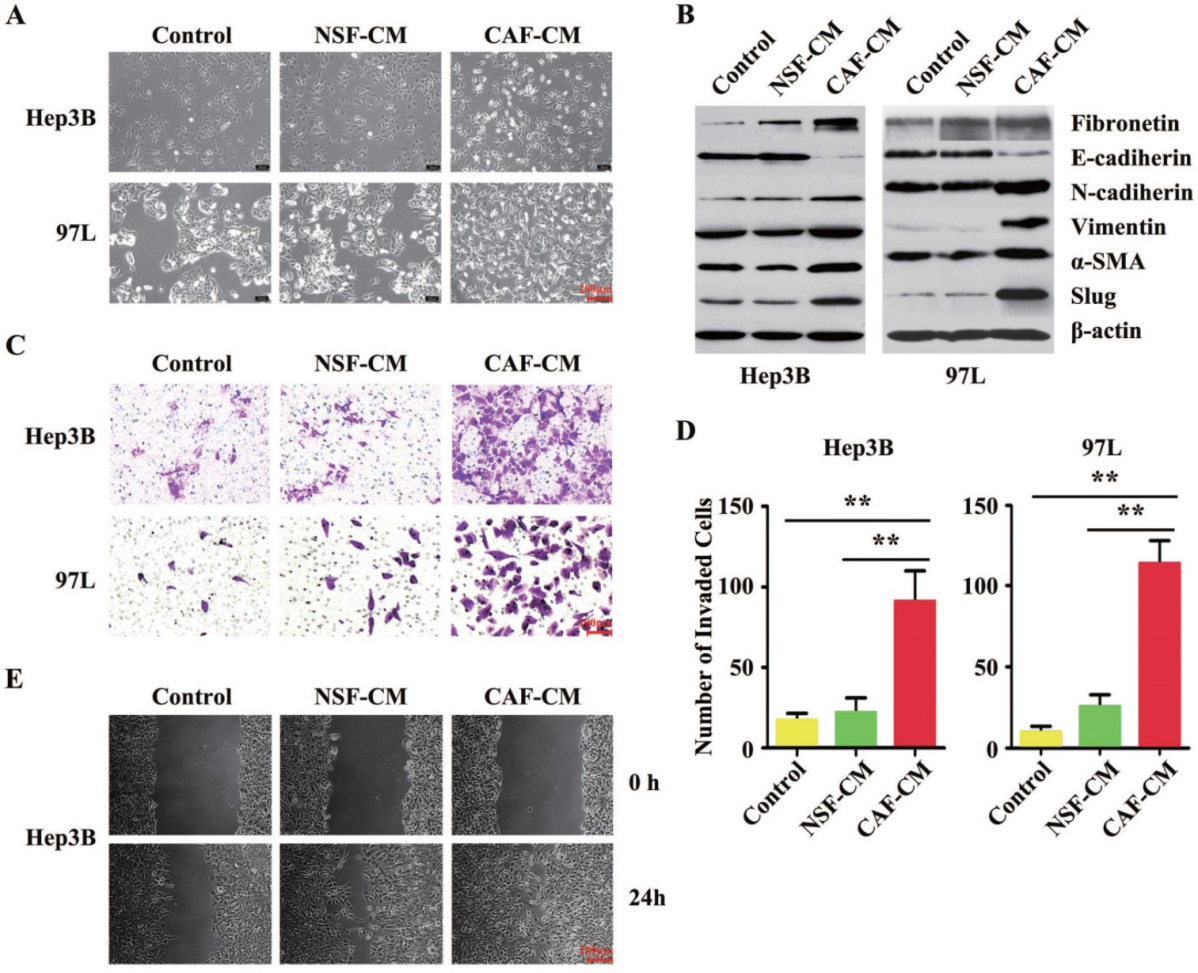
** H-CAFs promote HCC invasion and migration by inducing EMT.** (**A**) Microscopic images of HCC cells (Hep3B and 97L) co-cultured with NSF-CM or CAF-CM for 2-4 days. (**B**) Western blotting analysis shows that Hep3B and 97L exhibit a significant decrease in the expression of E-cadherin and a corresponding increase in the levels of fibronectin, N-cadherin, vimentin, α-SMA and Slug after treatment with CAF-CM. β-actin was used as a loading control. (**C**) Representative images and analysis show that the invaded cell s of Hep3B and 97L cells with or without CM treatment. The statistical analysis was shown in (**D**). The error bars represent ± SEM, ** *P*<0.01. Original magnification, 200×. (**E**) Microscopic observation of the areas between scratch fronts was recorded 0 and 24 h after scratching the surface of a confluent layer of HCC cells (Hep3B). NSF-CM, normal skin fibroblast conditional medium. CAF-CM, CAFs conditional medium.

As suggested by the clinical data, we further investigated the impact of CAFs on HCC cell biology *in vitro*. The established interaction model between H-CAFs and HCC cells 10 was used to investigate the underlying mechanism of H-CAF-promoted cancer progression. Compared to the negative controls or cells treated with normal skin fibroblasts conditional medium (NSF-CM), both Hep3B and MHCC-97L cells co-cultured with H-CAFs or receiving CAFs conditional medium (CAF-CM) treatment underwent changes from an agminated growth structure and epithelium-like morphology to a scattered growth structure and fibroblast-like morphology (**Figure 2A**), indicating the occurrence of EMT. For confirmation, the expression levels of EMT markers were analyzed in HCC cells treated with or without CAF-CM. In contrast to the controls or cells treated with NSF-CM, the expression of the epithelial marker E-cadherin in the cancer cells significantly decreased upon treatment with H-CAFs, whereas the expression levels of the mesenchymal markers N-cadherin, vimentin, fibronectin, α-SMA, and Slug increased (**Figure 2B**). Notably, EMT was not induced by treatment with the CM of the inactivated NSF.

To evaluate the changes in the invasion and metastasis capabilities of HCC cells treated with H-CAFs, Matrigel invasion and wound-healing assays were performed. The invasion assay revealed that the invasion capability of both HCC cell lines cultured with CAF-CM was markedly stronger compared with that of untreated cells or of cells cultured with NSF-CM (**Figure 2C and D**). The wound-healing assay demonstrated that CAF-CM significantly increased the motility and migration of HCC cells compared with NSF-CM or null CM (**Figure 2E**). Together, CAFs induced EMT in HCC cells *in vitro*.

### IL-6 and HGF were the key cytokines involved in H-CAF-mediated EMT

**Figure 3:**
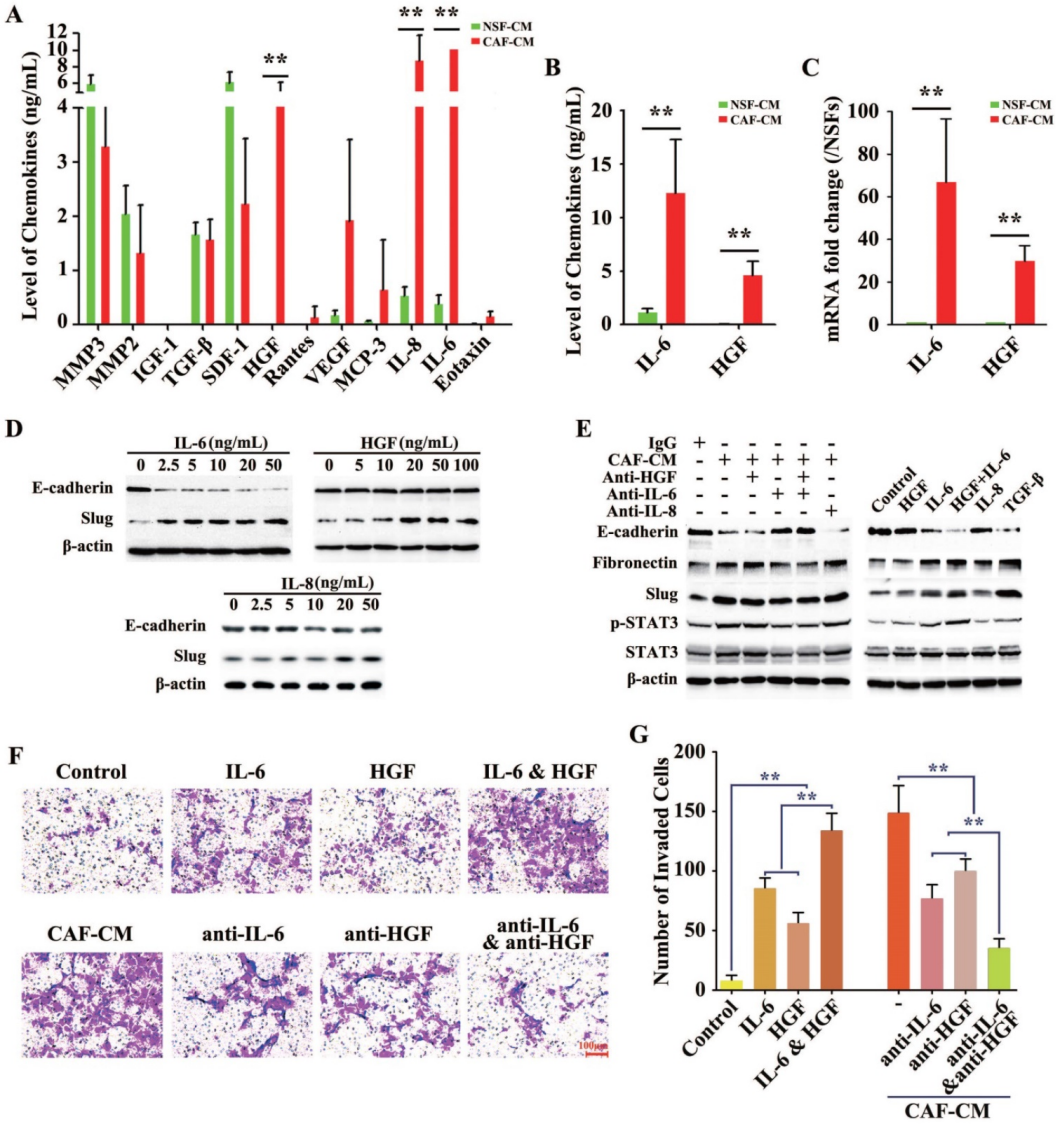
**Secretion of cytokines by differently derived fibroblasts (H-CAFs and NSFs).** (**A**) A liquid chip assay analysis indicated that the concentrations of these cytokines (IL-6, IL-8, MCP-3, HGF and VEGF) were significantly higher in the CM of H-CAFs than in the CM of NSFs. However, NSFs secreted a higher level of these cytokines (SDF1, MMP2 and MMP3) than the other three fibroblast types. TGF-β secretion was similar among the four fibroblast types. (**B**) The levels of IL-6 and HGF in the H-CAF CM were further verified by ELISA. (**C**) RT-PCR was used to detect the expression of *IL-6* and *HGF* in H-CAFs. (**D**) Recombinant IL-6, HGF or IL-8 was added to treat HCC cells with a dose dependent manner. Indicated antibodies were used to detect the protein level E-cadherin or Slug. (**E**) Left panel, the corresponding neutralizing antibodies were added to medium after HCC cells were treated with CAF-CM. Right panel, HCC cells were treated with recombinant IL-6, HGF or IL-8. Indicated antibodies were used to test the signals E-cadherin, Fibronectin, Slug, pSTAT3-S727 and STAT3. (**F and G**) Representative images and analysis show that the IL-6 significantly induced Huh7 cells invasion *in vitro*. Original magnification, 200×. The error bars represent ± SEM, * *P*<0.05; ** *P*<0.01.

The previous results indicated that certain molecules in the CAF-CM but not in the NSF-CM acted as the EMT-inducer. The concentrations of a total of 72 cytokines and chemokines were further measured in the CAF-CM and NSF-CM by secretomic assay using a suspension array system. The levels of several inflammatory cytokines and growth factors including IL-6, IL-8, monocyte chemoattractant protein 3 (MCP-3), vascular endothelial growth factor (VEGF), and HGF were significantly higher in the CAF-CM than in the NSF-CM, and IL-6, IL-8, and HGF were the most dramatically elevated, with the IL-6 level in the CAF-CM exceeding the examinable threshold (**Figure 3A**). Furthermore, ELISA (**Figure 3B**) and RT-PCR (**Figure 3C**) were performed to measure the protein and gene levels of IL-6 and HGF in the CMs, and showed results consistent with those of the secretomic assay.

Subsequently, to explore whether the EMT was induced by the above three markedly increased factors, we used human recombinant cytokines instead of CAF-CM to delineate the single or synergistic action of these specific cytokines in EMT induction. Recombinant IL-6 (rIL-6) potently and dose-dependently downregulated E-cadherin expression and upregulated Slug expression in HCC cells, even at a low concentration of 2.5 ng/mL. While Slug was increased by treatment with human recombinant HGF (rHGF) or recombinant IL-8 (rIL-8), E-cadherin levels did not obviously change in HCC cells treated with up to 100 ng/mL rHGF or up to 50 ng/mL rIL-8 (**Figure 3D**).

To further confirm the roles of IL-6, IL-8, and HGF in H-CAF-induced EMT, neutralizing antibodies to IL-6 (anti-IL-6), IL-8 (anti-IL-8), and HGF (anti-HGF) were administered individually or simultaneously to antagonize the effect of these secreted factors in the CAF-CM. The EMT induced by CAF-CM was largely reversed by blocking IL-6 in the CAF-CM, with the expression levels of E-cadherin, fibronectin, and Slug reverted nearly to the baseline levels. However, anti-IL-8 or anti-HGF alone did not markedly rescue the CAF-CM-induced EMT. Moreover, the EMT was almost completely reversed when both anti-IL-6 and anti-HGF were simultaneously added to the CAF-CM. The phosphorylation and activation of STAT3, a downstream molecule of the IL-6 and HGF signaling, is a key procedure during EMT 3. The level of p-STAT3 markedly increased when an EMT occurred in HCC cells (**Figure 3E**,** left panel**).

With TGF-β, a well-known EMT-inducer 3, as the positive control, rIL-6 but not IL-8 or rHGF obviously induced EMT. Interestingly, the downregulation of E-cadherin and upregulation of fibronectin and Slug in HCC cells were stronger in combined treatment with rIL-6 and rHGF than single treatment with rIL-6, suggesting a synergistic role of rHGF in rIL-6-induced EMT (**Figure 3E, right panel**).

Next, matrigel assay was applied to evaluate the roles of IL-6 and HGF secreted by H-CAFs in promoting HCC cell invasion. IL-6 alone or together with HGF significantly enhanced tumor invasiveness. Furthermore, anti-IL-6 or anti-HGF attenuated the CAF-CM-promoted invasion. When both neutralizing antibodies to IL-6 and HGF were simultaneously added to the CAF-CM, the invasive ability of tumor cells was most strongly inhibited (**Figure 3F and G**).

In the HCC sample, the relation of H-CAFs density with E-cadherin or with p-STAT3 were further testified by IHC, as showed in **Figure S1A** and **S1B**, respectively, E-cadherin expression levels were higher in the H-CAFs*^low^* subgroup than in the H-CAFs*^moderate/high^* subgroup. Consistently, E-cadherin IHC score in H-CAFs*^low^* subgroup was higher than H-CAFs*^moderate/high^* subset (*P*=0.025, **Figure S1C** and **S1D**). Moreover, high H-CAFs density correlated with overexpression of p-STAT3, and p-STAT3 IHC score of low density group were significantly higher than moderate and high density groups (*P*=0.043, **Figure S1E** and **S1F**). In addition, we used western blotting to test the total level of IL6R, STAT3 and p-STAT3 (**Figure S2A**), which showed a consistent result with Figure S1. And transwell assay of Hep3B, treated with H-CAFs*^low^*or H-CAFs*^moderate/high^*, was performed. We observed a higher capacity of cell migration after H-CAFs*^moderate/high^* treatment than H-CAFs*^low^* treatment, indicating that the secretions, especially cytokines could enhance the cell migration (**Figure S2B**).

Together, these data suggested the function of the signaling of IL-6 and HGF secreted by H-CAFs as prerequisite for the enhanced invasion and migration potencies during H-CAF-mediated EMT in HCC cells.

### Quantitative proteomic analysis revealed that TG2 expression was significantly elevated in HCC cells undergoing IL-6-induced EMT

We further investigated the intracellular molecular mechanism during CAF-induced EMT in HCC cells, and the differences in various protein levels before and after EMT was analyzed using a proteomics assay. To ensure accurate quantification and statistical assessment of the protein abundance changes, three replicate cultures of each treatment were used in this proteomics analysis using the 2-D DIGE technology combined with MALDI-TOF/TOF MS analysis. IEF strips with a broad pH range (3.0-10.0) were initially used for the 2-D DIGE experiment. IEF strips with pH 4.0-7.0 where significant changes in protein expression mostly located were then used for the 2-D DIGE experiment. Across all the gels, about 2,300 protein spots with quantitative differential expressions in HCC cells before and after EMT were repeatedly detected. After the DIGE image analysis with the DeCyder software and protein identification using the acquired MALDI-TOF/TOF data, candidates of EMT-related proteins were screened out. A total of 36 spots with >1.5 folds changes in expression were identified, and MS analysis further confirmed 16 unique proteins (**Table 3**).

**Figure 4:**
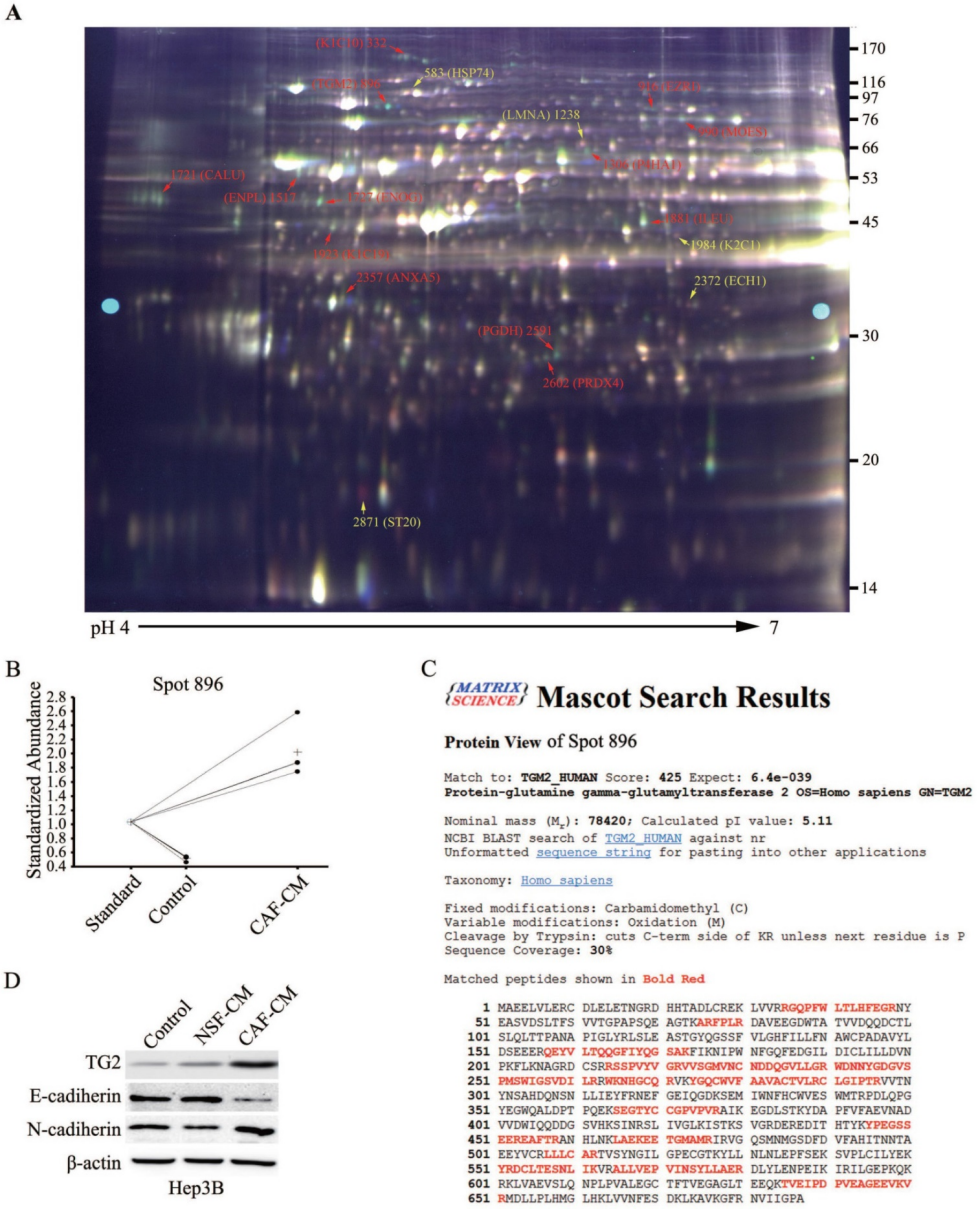
** The total proteins from the HCC cells with or without EMT were extracted and applied 2-D DIGE and MALDI-TOF/TOF MS analysis.** (**A**) A 2-D DIGE pattern (Gel 4). According to the experimental design, protein samples from each HCC cultures were extracted and labeled with CyDyes, Cy2-labeled internal standard sample (pool), Cy3-labeled sample (EMT HCC) and Cy5-labeled sample (HCC) were mixed and separated in a 2-D DIGE gel (IEF on pH 4-7 strips followed by 12.5% second dimension SDS-PAGE), the DIGE gel was then visualized with a Typhoon 9400 scanner. Marked protein spots indicate the significantly expressed proteins which corresponding to the proteins listed in Table 3. (**B**) A typical example is the protein spot 896, whose abundance increased by 4.27 folds after EMT. (**C**) LC-MS analyzed spot 896 and identified the protein sequence of peptides extracted from spot 896. (**D**) Western blotting analysis confirmed TG2 expression level in Hep3B, 97L or Huh7 cells after NSF-CM or CAF-CM incubation.

A representative 2-D DIGE gel is shown in **Figure 4A**. The red spots were proteins with significantly increased expression after EMT, and the yellow spots indicated significantly downregulated proteins. Among them, a typical example is the protein spot 896, whose abundance increased by 4.27 folds after EMT (**Figure 4B**). MS data analysis revealed that spot 896 was TG2, which has been previously reported to be associated with EMT in other tumor entities 16, and the corresponding PMF data are detailed in **Figure 4C**. Similar to the identification of TG2, a total of 10 other protein spot changes on the 2-D DIGE gel were found that might be associated with EMT in HCC cells (**Figure 4A** and **Table 3**). Eight unique proteins were putatively upregulated and two downregulated, respectively, during HCC cell EMT. Typical examples of upregulated proteins were cytokeratin (CK)-10 (spot 322), CK-19 (spot 1923), Meosin (spot 990) and Ezrin (spot 916). Of these proteins, CK-10 17, CK-19 18, and Ezrin 19 have been reported to exert significant function during EMT, supporting the reliability of the proteomics screening.

Among the 16 proteins, TG2 attracted our attention most and became the focus of our further research because its expression increased most remarkably by 4.27 folds after HCC cells underwent EMT stimulated by CAF-CM (**Table 3**). To further validate the proteomic results and to confirm the involvement of the potential candidate molecule with differential expressions in HCC cell EMT, western blotting comparative analysis was performed, and showed that TG2 was significantly upregulated during CAF-induced EMT in Hep3B, 97L, and Huh7 cell lines after treated with the CAF-CM for 48 h (**Figure 4D and Figure S3**). Notably, NSF-CM also markedly increased the expression of TG2 in 97L and Huh7 cells. Together, these findings strongly supported the involvement of TG2 in EMT.

### TG2 is a crucial regulator in CAF-induced EMT in HCC cells

**Figure 5:**
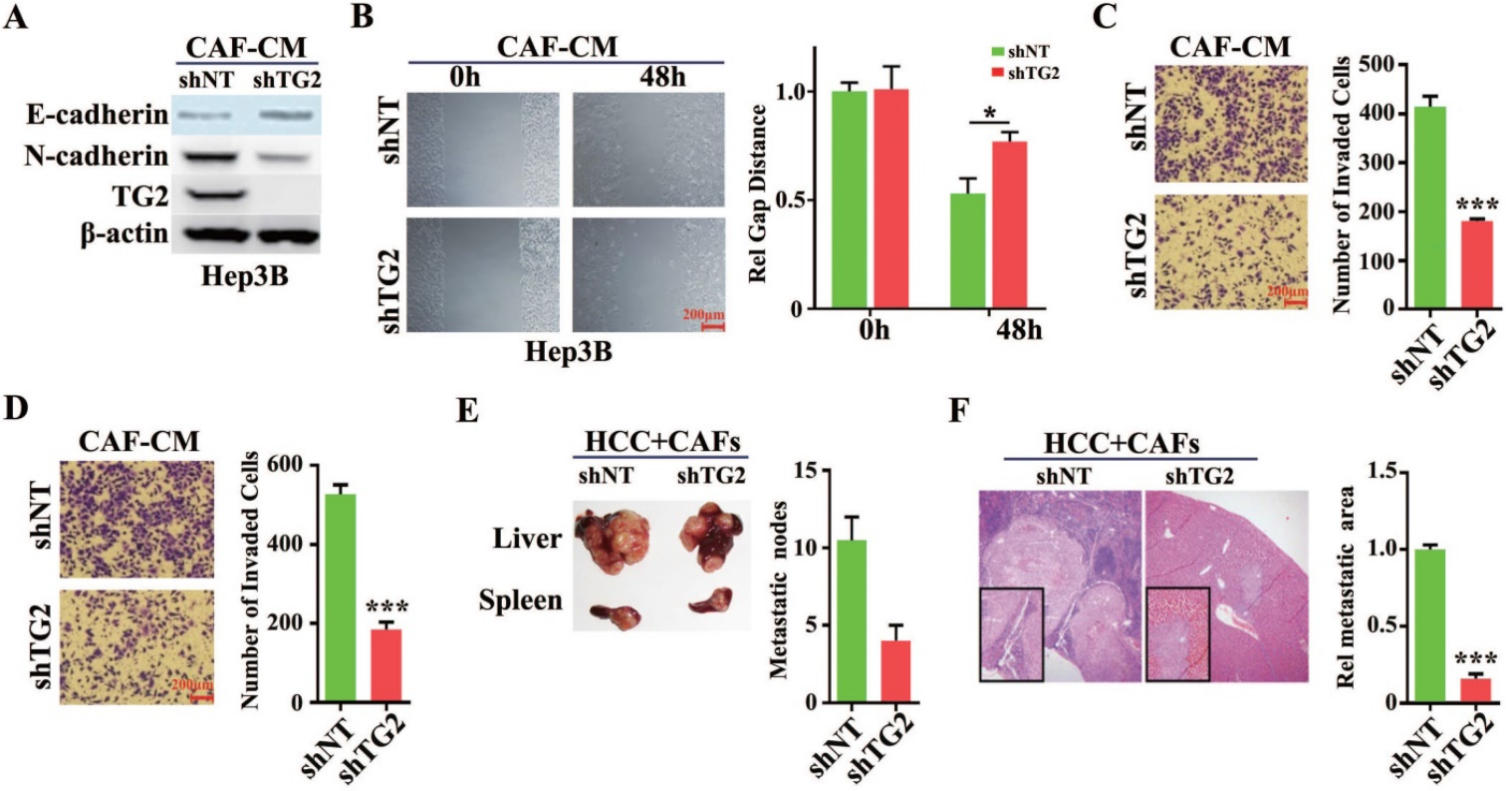
**TG2 was required for CAF induced EMT of HCC cells.** (**A**) TG2 was stably knocked down by specific small hairpin RNA (shRNA) in Hep3B cells. A non-target (NT) shRNA was used as a control. N-cadherin and E-cadherin were used as indicators of EMT initiation. (**B**) Wound healing, (**C**) transwell, and (**D**) invasive assays were performed using Hep3B- shNT and shTG2 cells. The error bars represent ± SEM; * *P*<0.05, *** *P*<0.01). (**E**) In the nude mouse metastatic tumor model, CAFs and Hep3B- shNT or shTG2 cells (1:1) (HCC + CAFs) were co-injected into the spleen of nude mice. After 21 days, the tumor of liver metastases was counted and then confirmed by H&E staining (**F**). Original magnification, 50×.

To validate whether TG2 was involved in the regulation of HCC cell EMT, a small hairpin RNA (shRNA) targeting human *TG2* or lentivirus introduced overexpressing TG2 was transfected into HCC cells. Western blotting analysis showed that TG2 was remarkably depleted in Hep3B cells and E-cadherin protein was increased while N-cadherin was decreased after transfection of shTG2 (**Figure 5A**). And TG2 was dramatically enhanced in Huh7 cells when lentivirus infected after 72h and E-cadherin protein was decreased while N-cadherin was increased (**Figure S4A**). A wound healing assay showed loss of TG2 in Hep3B cells impaired their cell migration even under CAF-CM stimulation (**Figure 5B**). When stably expressed TG2 in Huh7 cells, we observed obviously raised efficiency of migration after scratch (**Figure S4B**). In the *in vitro* model of CAF-CM induced EMT, transwell and invasion assays demonstrated that the migratory and invasive abilities of Hep3B cells were significantly reduced after transfection with shTG2 compared with transfection with control (**Figure 5C and 5D**). However, overexpression of TG2 in Huh7 significantly improved the migration and invasion of Huh7 cells even without co-incubation with CAF-CM (**Figures S4C and S4D**). In the nude mouse metastatic tumor model, CAFs and HCC cells (1:1 ratio) were co-injected into the spleen of nude mice, and liver metastases of HCC cells were observed. When TG2 was silent in HCC cells, the number and volume of liver metastases were significantly reduced (**Figure 5E and 5F**). After high expression of TG2, the metastases of Huh7 cells were significantly increased from spleen to liver in nude mice (**Figures S4E and S4F**). Therefore, we can conclude that TG2 plays an important role in CAF-induced EMT of HCC cells.

### Expression of TG2 in HCC cells is associated with IL-6/IL6R/STAT3 axis

**Figure 6:**
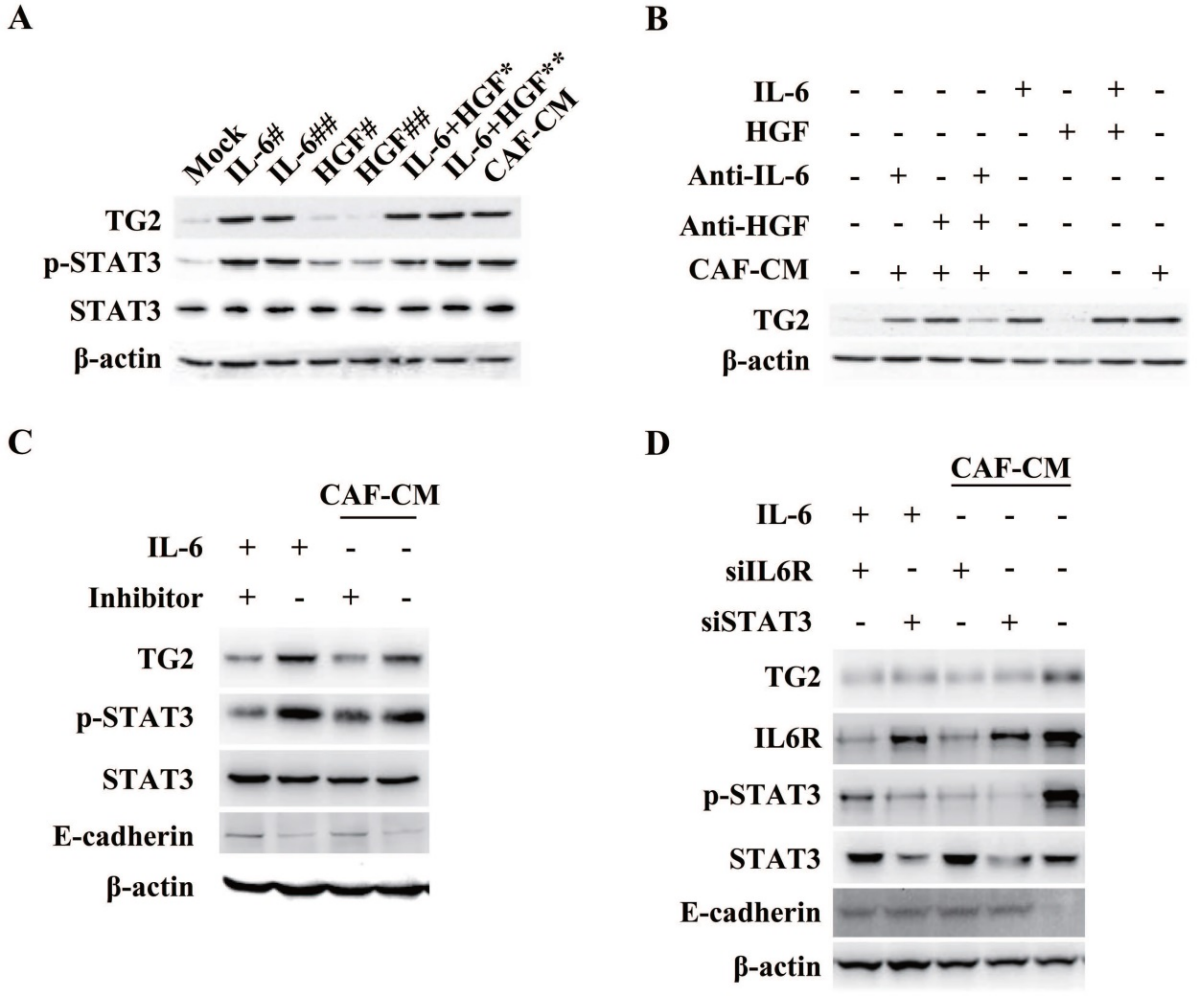
**Expression of TG2 in HCC cells is associated with IL-6/IL6R/STAT3 axis.** (**A**) Recombinant IL-6 or HGF was added to the medium to treat Hep3B cells with a dose dependent manner. Indicated antibodies were used to test the protein level pSTAT3-S727, STAT3 and TG2. #, 5ng/mL; ##, 10ng/mL; *, IL-6 (5 ng/mL) + HGF (5 ng/mL); **, IL-6 (10 ng/mL) + HGF (10 ng/mL). (**B**) The corresponding neutralizing antibodies were added to medium after HCC cells were treated with recombinant IL-6 or HGF, independently. Indicated antibodies were used to test the protein level of TG2. (**C**) IL6R inhibitor, Tocilizumab (1 µM) was added to the medium and incubated for 12 hr. Indicated antibodies were used to test the protein levels of TG2, pSTAT3-S727, STAT3 and E-cad. (**D**) Small interference RNA target IL6R or STAT3 was used to deplete endogenous *IL6R* or *STAT3* genes. Indicated antibodies were used to test the protein levels of TG2, IL6R, pSTAT3-S727, STAT3 and E-cadherin.

Among the EMT-related significant differential proteins screened by proteomic analysis, we found that the expression of TG2 was the most significant (4.27-fold). Further, we will demonstrate the correlation between TG2 expression and CAF-induced EMT. As shown in **Figure 6A**, the addition of human recombinant IL-6 to HCC induced an increase in TG2 expression, and the expression level of TG2 was consistent with the serine 727 phosphorylation level of STAT3 (pSTAT3-S727), whereas the addition of different doses of human recombinant HGF could not induce pSTAT3-S727 and TG2 expression. The addition of IL-6 neutralizing antibody to the CAF-CM-induced EMT of HCC cells significantly inhibited TG2 expression, whereas the addition of HGF neutralizing antibody had no significant effect on TG2 expression (**Figure 6B**). In order to identify that the expression of TG2 in HCC cells is regulated by IL-6/IL6R/STAT3 axis, we added Tocilizumab to HCC cells and found that pSTAT3-S727 is down-regulated with the addition of Tocilizumab and TG2 expression levels were also down-regulated (**Figure 6C**). Further, we used shRNA to interfere with the expression of IL6R and downstream signaling molecule STAT3 in HCC cells, respectively, as shown in **Figure 6D**, both of which significantly affected TG2 expression. These results strongly suggest that the expression of TG2 is mainly regulated by the IL-6/IL6R/STAT3 axis.

### STAT3 trans-activated TG2 gene expression to promote IL-6-induced EMT in HCC

**Figure 7:**
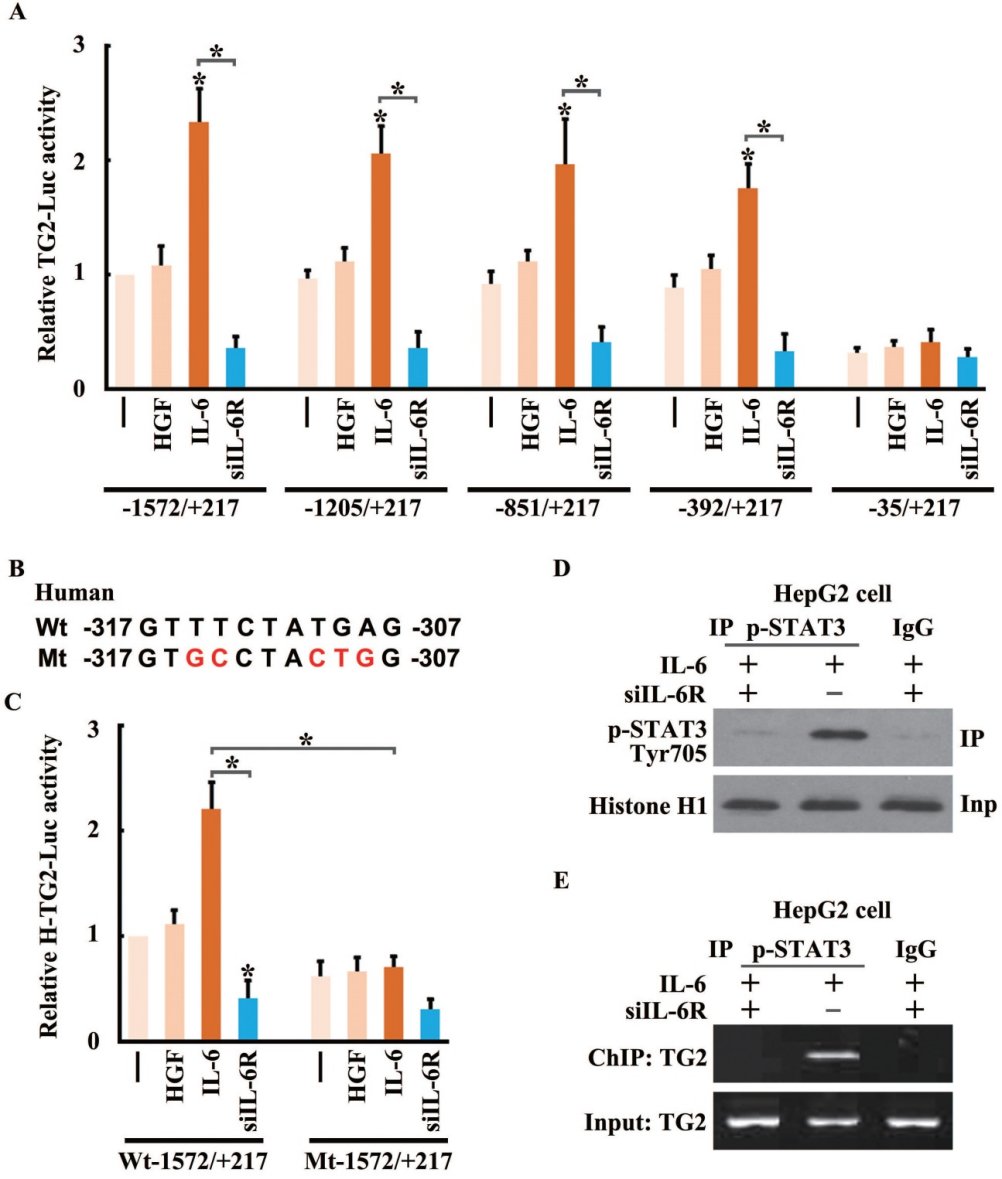
**STAT3 trans-activates *TG2* gene expression to promote IL-6-induced EMT.** (**A**) Human *TG2* gene promoter (NC_000020.11) containing -1572/+217 was cloned and then truncations of four subclones: -1205/+217, -851/+217, -392/+217 and -35/+217 in pGL3B were used to construct the reporter plasmids of TG2 (TG2-Luc), and performed luciferase reporter assay. (**B**) A conserved STAT3 binding motif was located in the region of -317 to -307. Mutagenesis was performed to destroy STAT3 potential binding site. (**C**) Luciferase reporter assay was performed using wild type and mutant *TG2* gene promoter. (**D**) Co-immuno-precipitation of pSTAT3-S727 and histone H1 was performed. (**E**) Chromatin-immuno-precipitation of pSTAT3-S727 targeting TG2 promoter was performed.

To confirm whether the IL-6/STAT3 signaling could stimulate the transcription of TG2, we cloned the Human *TG2* gene promoter (NC_000020.11) containing -1572/+217 base pairs of human *TG2* promoter 20 to construct the reporter gene of *TG2* (TG2-Luc), and performed luciferase reporter gene assay. Culturing HCC cells in IL-6- but not in HGF-containing media led to an approximately threefold increase in *TG2* promoter activity, and the increased activity was suppressed by IL-6 receptor (IL6R) siRNAs (**Figure 7A**), supporting that* TG2* was transcriptionally upregulated by the IL-6/STAT3 signaling during EMT.

To determine whether STAT3 directly regulated the activity of the *TG2* gene promoter, the core sequences for *TG2* promoter activation were identified. A series of reporter constructs containing deletions of the *TG2* promoter 5'-flanking region was cloned into the reporter vector. All of the truncated *TG2* promoter constructs had the same 3'-end. One of the deletion constructs (nucleotides -392 to +217) showed the same activity as the original construct (nucleotides -1,572 to +217) in the IL-6-containing media, whereas in another deletion construct (nucleotides -235 to +217), the increased promoter activity was almost completely abolished (**Figure 7A**). Thus, the principal *TG2* transcriptional control element was narrowed down to a region spanning nucleotides from -392 to -35.

Promoter analysis identified a potential STAT3-binding site between nucleotides -317 and -307 (5'-GTTTCTATGAG-3') in the core region of the *TG2* promoter. To evaluate the role of this putative binding site in *TG2* promoter activation, mutations of the putative site were introduced into the reporter vector (**Figure 7B**), and markedly disrupted the activation of the *TG2* promoter in response to IL-6 treatment (**Figure 7C**). To confirm that STAT3 and the *TG2* promoter interacted *in vivo*, a ChIP assay was performed for HCC cells treated with IL-6 or IL-6R siRNA. Chromatin was precipitated by a p-STAT3 (Ser727) antibody. The p-STAT3 (Ser727) protein, crosslinked to DNA, was readily detected in IL-6-treated cells (**Figure 7D**). Precipitated DNA was assayed using PCR with primers flanking the STAT3-binding site of the *TG2* promoter. The DNA fragment was amplified strongly in IL-6-treated cells (**Figure 7E**).

## Discussion

**Figure 8:**
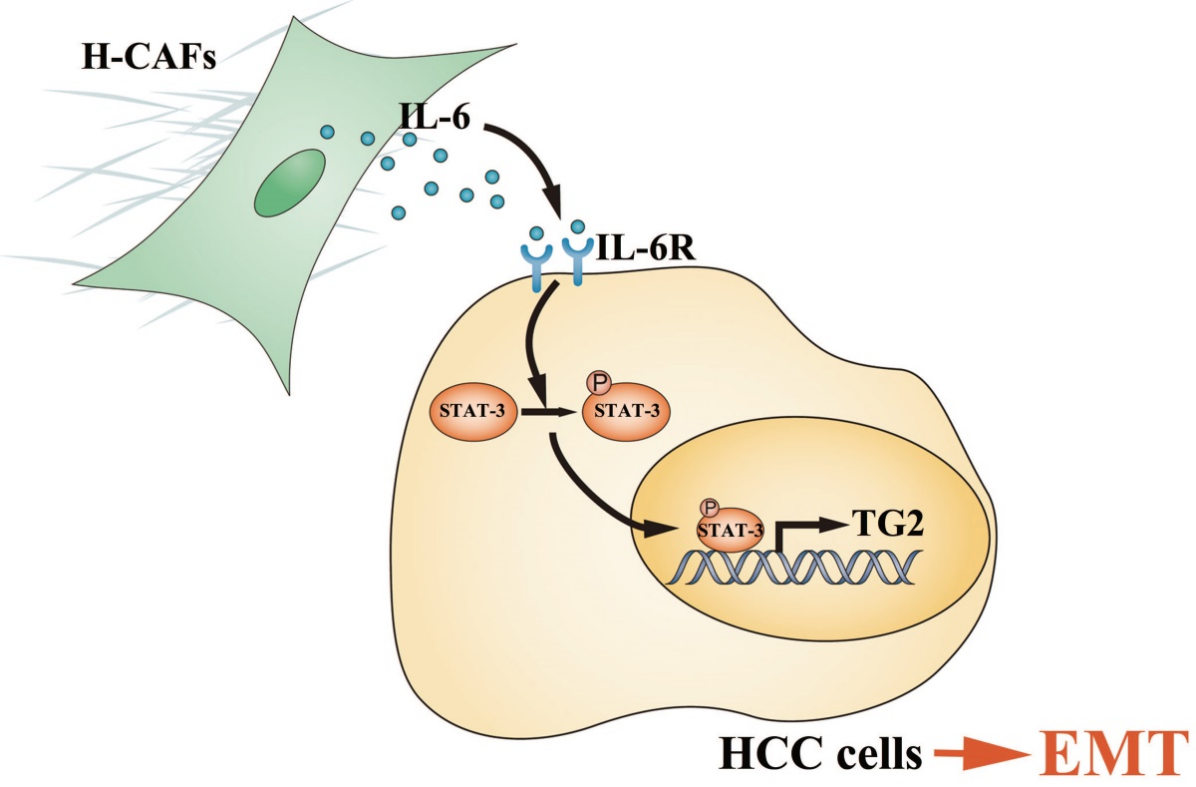
Schematic diagram showing that H-CAF secreted IL-6 was received by HCC cells and stimulated IL-6/IL6R/STAT3 axis. In turn, the activated STAT3 targeted the promoter of *TG2* gene and promoted TG2 expression which consequently initiates EMT.

Primary HCC is one of the most deadly human cancers and has a very poor prognosis. Most of HCC patients die from intrahepatic and distant metastases 21. Therefore, a better understanding of HCC metastatic mechanism and the exploration of novel treatment strategies are urgently needed. An increasing number of studies have highlighted the contribution of stromal cells to tumor metastasis, and CAFs are the most prominent cell type with the widest distribution in the tumor stroma of many cancers, most notably breast, prostate and pancreatic carcinomas 6, 22, 23. However, the crosstalk and underlying mechanism between HCC cells and CAFs are poorly understood. In this study, we demonstrated that H-CAFs secreted IL-6 was received by HCC cells and stimulated the IL-6/IL6R/STAT3 axis. In turn, the activated STAT3 targeted the promoter of *TG2* gene and promoted TG2 expression which consequently initiates EMT (**Figure 8**).

Firstly, we reported here that 72.6% of HCC samples in our study were rich in H-CAFs, and a Kaplan-Meier analysis showed that HCC patients with a high density of H-CAFs generally had a worse prognosis than those with a low H-CAFs density. Importantly, a multivariate Cox regression analysis indicated that the H-CAFs density was an independent prognostic factor for the OS and PFS. Specifically, our results revealed that a higher density of H-CAFs correlated with EMT, which was identified by a low expression of E-cadherin. Moreover, the loss of E-cadherin closely correlated with a shorter PFS, although it was not an independent indicator of PFS. These observations indicate that H-CAFs may promote tumor invasion and metastasis via EMT in HCC by regulating the E-cadherin-mediated cell-to-cell adhesion, which was consistent with the results of our *in vitro* experiment.

Secondly, in this study, the cytokines secreted by H-CAFs, NSFs, and HCC cells were screened using a high-flux assay, and we found that CAFs released an abnormal amount of inflammation-related factors and growth factors, such as IL-6, IL-8, VEGF, MCP-3, TGF-β, SDF-1 and HGF. A larger variety of soluble factors have been proposed as the key mediators of the crosstalk between malignant cells and CAFs 24-27. Further, we confirmed that IL-6 and HGF secreted by H-CAFs but not by the liver cancer cells were crucial to EMT of HCC cells. Hence, we highlighted the importance of a paracrine effect on HCC cells by H-CAFs, rather than autocrine function. IL-6, a cytokine that can be secreted by various cell types, such as monocytes, macrophages, fibroblasts, endothelial cells, immune cells, and hepatocytic liver cancer progenitors 28, 29, was identified as a pleiotropic inflammatory cytokine and involved in the regulation of acute and chronic inflammation, which are closely related to HCC progression 30. Previous studies have reported that excessive IL-6 production by macrophages in response to carcinogen-mediated tissue damage was an important determinant of a higher susceptibility to HCC in male mice, which indicated that IL-6 plays an essential role in the initiation of liver carcinogenesis 31. In this study, we found that H-CAFs appear to be the predominant producers of IL-6, because the concentration of secreted IL-6 was significantly higher in H-CAFs CM than in HCC CM.

We previously found that H-CAFs-derived HGF promoted HCC cell proliferation 10. However, in this study, we proved that HGF is not an important cytokine that contributes to EMT of HCC. TGF-β was previously reported to be the mediator of tumor proliferation, migration, and invasion 32, 33, but in our study, the concentration of TGF-β is in low level and did not differ between H-CAFs and NSFs, which indicated that TGF-β may probably playing a weaker role in H-CAFs-triggered EMT. In addition to TGF-β and SDF-1, other types of inflammatory mediators, such as, IL-8, MCP-3, etc., were secreted by H-CAFs at high levels, but all these possibilities require further study.

Thirdly, we discovered that the H-CAFs secreted IL6 positively correlated with p-STAT3 signal in HCC cells. And a high transcriptional activity of STAT3 was found to positively associate with TG2 expression in HCC cells. Beyond its roles in inflammation, TG2 has been proven to promote EMT in several types of cancer 34-36. Further, we identified the essential role of TG2 in EMT with both *in vitro* and *in vivo* methods, such as transwell, invasion and mouse metastatic tumor model. To our knowledge, this is the first report demonstrating that TG2 promotes cancer cell metastasis in HCC. Besides, a previous proteomic study showed that TG2 is a potential biomarker for HCC 37. Thus, our study further highlights the diagnostic and therapeutic potential of TG2 in HCC. The JAK/STAT3 axis is reportedly activated by many inflammatory signals 38. We first utilized the specific inhibitor of IL6R to frame the signaling pathway referred to TG2 expression. Then we proved IL6R/STAT3 axis in HCC cells was required for TG2 expression by depleting IL6R or STAT3. By layer-by-layer approach, we confirmed that the IL-6/IL6R/STAT3 axis is required for HCC cells to regulate TG2 expression.

## Materials and Methods

### HCC samples

A consecutive series of 124 patients with histologically confirmed HCC who were admitted to The Third Affiliated Hospital of Sun Yat-sen University (Guangzhou, China) and who underwent radical hepatectomy from November 2003 to January 2008 was included. The median age was 50 (range, 22 to 73) years. Patients were included if they met the following criteria: (a) before operation, they had not received any anticancer therapy (e.g., trans-catheter arterial chemoembolization (TACE), chemotherapy, and radiotherapy), (b) the corresponding paraffin-embedded specimens were well preserved, and (c) the follow-up information was complete. Paraffin-embedded specimens and clinical data were retrieved for all patients. The samples were anonymously coded in accordance with the local ethics guidelines as stipulated by the Declaration of Helsinki 39. Our study was approved by the Clinical Research Ethics Committee of The Third Affiliated Hospital of Sun Yat-sen University, and written informed consent was obtained from each participant.

Tumor stage was defined according to the 2010 AJCC/UICC TNM staging system for HCC. Patients were followed-up until January 2014. Overall survival (OS) was defined as the time from surgery to death of any cause or last follow-up and progression-free survival (PFS) was the time interval between surgery and local failure, distant metastasis, death, or last follow-up.

### Immunohistochemistry analysis

The paraffin-embedded tissue sections (4 µm thick) were prepared according to classical methods, and immunohistochemistry (IHC) staining was performed as previously described 10. The tissue microarray slides were incubated at 4°C overnight with primary antibodies. The brown granules in the cytoplasm or nuclei were regarded as positive staining. Non-immune serum was substituted for the primary antibodies to serve as a negative control. Two independent pathologists evaluated the immunostaining results according to the product or sum of the intensity and stain area score without knowledge of the clinical data of patients. The intensity of staining was scored as follows: score 0, negative; score 1, borderline staining; score 2, weak staining; score 3, moderate staining; and score 4, strong staining. We scored the stain area according to the percentage of positive-stained cells in the field: score 0, negative; score 1, 0-25%; score 2, 25-50%; score 3, 51-75%; and score 4, 76-100%. The density of H-CAFs was identified by the α-SMA immunoreactivity and classified into two patterns (high or low) according to previously described criteria 10, 40. Additionally, the density of H-CAFs, which was high, low or difficult to distinguish, was categorized as moderate.

### H-CAF isolation and conditioned medium (CM) collection

H-CAFs were isolated from the cancerous regions of the tumor samples from HCC patients, as described previously 10. Normal skin fibroblasts (NSFs) were obtained from the foreskin biopsies of healthy donors. Briefly, the tissues were minced and digested in Roswell Park Memorial Institute (RPMI) 1640 medium (Invitrogen, Carlsbad, USA) supplemented with 10% fetal bovine serum (FBS; Gibco-BRL, Grand Island, USA), 1 mg/mL type I collagenase (Sigma, St. Louis, USA), and 100 U/mL hyaluronidase (Sigma) at 37 °C for 6-8 h, washed twice with phosphate-buffered solution (PBS; Sigma), and then centrifuged at 450 g for 8 min. They were finally re-suspended in RPMI 1640 supplemented with 10% FBS, 100 IU/mL penicillin, and 100 mg/mL streptomycin, and then cultured at 37 °C in a humidified environment with 5% CO_2_. Differential trypsinization was applied during sub-culturing for selection of fibroblasts. The percentage of purified fibroblasts was ~95% after 2-3 passages, which was determined by immunofluorescence.

For collection of CM from H-CAFs (CAF-CM), the fibroblasts were plated in 75 cm^2^ flasks, washed twice with PBS 4 days later, and incubated for 48 h with serum-free Dulbecco modified Eagle medium (DMEM; Gibco-BRL). Then, the supernatant was harvested, centrifuged at 3,000 rpm for 5 min, passed through a sterile filtration system with a 0.45 mm PVDF membrane, and stored at -80 °C for use.

### Cell culture and treatment

The HCC cell lines Hep3B, HepG2, Huh7 (American Type Culture Collection, Manassas, USA) and MHCC-97L (Hepatocellular Carcinoma Center, Zhongshan Hospital, Shanghai, China), were cultured in Minimum Essential Medium (MEM; Gibco-BRL) supplemented with penicillin (100 U/mL), streptomycin (100 μg/mL), and 10% FBS. Cells were maintained in DMEM supplemented with penicillin (100 U/mL), streptomycin (100 μg/mL), L-glutamine (300 μg/mL), and 10% FBS. Cells were passaged at a ratio of 1:4 every 4-5 days.

To observe H-CAF-induced or cytokine-induced EMT, HCC cells at the logarithmic growth phase were first digested and then placed into plates (2×10^5^ cells/well). After 12-24 h, we added to each well DMEM plus 1 mL 10% FBS and 1 mL CAF-CM, or different concentrations of human recombinant IL-6 and HGF (PeproTech, Rocky Hill, USA), with or without IL-6 and HGF neutralizing antibodies (0.5 and 20 μg/mL, respectively; R&D, Minneapolis, USA). The culture media were renewed every day.

### Cell wounding, invasion, and migration assays

For wounding assay, as HCC cell confluence reached 90% after transfection, cells were wounded by generating a longitudinal scratch within variation under 5% using a robotically driven (Seiko) stainless-steel pin programmed to deliver a scratch of 0.75 × 4 mm.

After HCC cells were co-cultured with H-CAFs in a transwell system (Corning, New York, USA), or treated with CAF-CM as descripted above. Then HCC cells were lysed in prechilled lysis buffer, and 10 μg of protein from the supernatant was subjected to Western blotting, probed with antibodies of EMT markers, E-cadherin, N-cadherin, vimentin, and β-catenin (CST, Danvers, USA).

Cell invasion analysis was performed using a transwell system. Forty-eight hours after RNA interference, the filters coated with matrigel (Sigma, USA) in the upper compartments were applied with 2×10^5^ HCC cells, and the lower compartments were filled with 2×10^5^ H-CAFs, which were mixed with DMEM supplemented with 10% FBS. After 36 h, migrated cells on the bottom surface were fixed with methanol and counted after stained with Giemsa. For migration assay, the HCC cells were cultured as confluent monolayers for 48 h after treatment, and wounded by removing a 300 to 500 μm strip of cells across the well with a 200 μL pipette tip.

### H-CAF secretomic analysis

H-CAFs (2×10^5^/well) were plated into 6-well plates and cultured for 24 h, and the CAF-CM was collected with the secretion of totally 72 cytokines measured using Bio-Plex Pro™ Human Cytokine, Chemokine, and Growth Factor Assays with a Bio-Plex 200 suspension array system (Bio-Rad, Hercules, CA, USA) according to the manufacturer's instructions. The levels of HGF and IL-6 in the CM from fibroblasts obtained using the same method were verified using the human enzyme-linked immunosorbent assay (ELISA) kits (R&D Systems, Minneapolis, MN, USA) following the manufacturer's instructions.

### Two-dimensional (2-D) difference in gel electrophoresis (DIGE) analysis

2-D DIGE was performed as described previously 41. HCC cells with or without EMT induction were washed using ice-cold Tris-buffered sucrose solution (10 mM Tris, 250 mM sucrose, pH 7.0), scraped, and lysed in ice-cold lysis buffer (30 mM Tris-HCl, 7 M urea, 2 M thiourea, 4% (w/v) CHAPS, pH 8.5). Protein labeling was conducted with CyDye DIGE Fluors (GE Healthcare, UK) as described in the DIGE user manual. The protein sample was dissolved in rehydration buffer (7 M urea, 2 M thiourea, 4% (w/v) CHAPS, 40 mM dithiothreitol (DTT), 0.002% (w/v) bromophenol blue, 1% IPG buffer, pH 4.0-7.0). Rehydration and isoelectric focusing (IEF) were performed following the manufacturer's instructions using IPG strips (24 cm, pH 4.0-7.0) with an Ettan IPGphor II apparatus (Amersham Biosciences, Uppsala, Sweden). After IEF, the proteins were reduced and alkylated by successive treatments with equilibration buffer containing 2% (w/v) DTT followed by 2.5% (w/v) iodoacetamide for 15 min. The proteins were resolved in the second dimension on a 12.5% SDS-PAGE gel (24×20 cm) using an Ettan DALT Six instrument (Amersham Biosciences).

Gel images were acquired using a Typhoon 9400 scanner (Amersham Biosciences). The Cy2, Cy3, and Cy5 signals were individually imaged with excitation/emission wavelengths of 488/520, 532/580, and 633/670 nm, respectively. The 2-D DIGE gel images were analyzed using the DeCyder software (version 6.0; Amersham Biosciences). The protein expression patterns of HCC cells undergoing EMT were compared with those of the controls. Protein spots with significant differences in abundance (greater than 1.5 folds) were selected in the stained gels for spot picking.

### Matrix-assisted laser desorption/ionization (MALDI)-time of flight (TOF)/TOF mass spectrometry (MS) analysis

MS analysis was performed as previously described 41. Briefly, the Deep Purple-stained gels were subjected to an Ettan Spot Handling Workstation (Amersham Biosciences) for preparation of protein samples for MALDI-TOF/TOF MS analysis. Protein identification was performed using a MALDI-TOF/TOF MS system (Bruker Delton, Germany), and the peptide mass spectrum was obtained using the positive ion reflector mode. Monoisotopic peak masses were limited within the mass range of 1,000-4,000 Da, and the S/N minimum was set to five. Trypsin autolysis peptides of masses 842.5 and 2,211.1 were used as internal standards. Five of the most intense ion signals were automatically selected as precursors for MS/MS acquisition. Peptide mass finger printing (PMF)-combined MS/MS spectra were searched against the NCBInr database using the GPS Explorer (version 3.6; Applied Biosystems) and MASCOT (version 2.1; Matrix Science) software with the following parameters: human as taxonomy, trypsin cleavage, one missed cleavage allowed, carbamidomethylation as fixed modification, oxidation of methionines allowed as variable modification, peptide mass tolerance at 75 ppm, and fragment tolerance at 0.2 Da. Significantly high MASCOT scores that resulted in a confidence interval (CI) greater than 95% for either PMF and/or MS/MS data for a spot were considered as an indication of a credibly identified protein.

### Construction of expression and small interfering RNA (siRNA) lentiviral vectors

TG2-expressing plasmid was constructed by cloning the cDNA-encoding human TG2 (NM_004613) into the pCDH-CMV-MCS-EF1-copGFP vector (System biosciences, Mountain View, USA). The short hairpin RNA (shRNA) sequence for targeting human TG2 mRNA was 5'-AACTGCAGTGACTTTGACGTCTT-TTCAAGAGA-AAGACGTCAAAGTCACTGCTTTTTTC-3', containing sense-loop (TTCAAGAGA)-antisense-termination signal sequence, were inserted downstream of the U6 promoter of the lentiviral vector pLL3.7. The expression vectors were mixed with plasmids pGag/Pol, pRev, and pVSV-G, and transfected into 293T cells using Lipofectamine 2000 (Invitrogen). All constructs were confirmed by sequencing. Lentivirus infections were carried out in the presence of 5-10 μg/mL polybrene. For transfection of the HCC cells with different metastatic potentials, pairs of TG2 siRNAs and a negative-control mismatch sequence were transfected, and control cells were only transfected with empty vectors.

### Western blotting

Western blotting was performed with the lysates from indicated cultured cells and followed by the immunoblotting with corresponding antibodies. Briefly, after trypsinizing, the cells were harvested and washed twice with cold PBS. Cell pellets were resuspended and lysed in the buffer (50 mM Tris-HCl, pH 7.4, 150 mM NaCl, 1% Triton X-100, 5 mM EDTA, 1 mM NaVO_3_, 50 mM NaF and protease inhibitor cocktail). Then the lysates were centrifuged at 13,000 g to remove the cell debris. Supernatant was resolved on SDS-PAGE gel and subsequently transferred to PVDF membranes (GE). The blots were blocked with 5% non-fat milk followed by the incubation of primary antibodies and HRP-conjugated secondary antibodies. Blots were developed using SuperSignal West Pico (Thermo Scientific, USA) and detected by a ChemiDoc MP Imaging Workstation (Bio-Rad).

### Luciferase reporter gene assay

To determine the TG2 promoter region, luciferase reporter gene assay was performed with the Dual-Luciferase Reporter Assay System (Promega, Madison, USA). Human TG2 gene promoter (NC_000020.11) containing -1572/+217 base pairs of human TG2 promoter 20 was applied to construct the reporter gene of TG2 (TG2-Luc). A series of reporter constructs containing deletions of the TG2 promoter 5'-flanking region (-1572/+217, -1205/+217, -851/+217, -392/+217, and -35/+217) were cloned into the reporter vector. HepG2 was co-transfected with TG2 promoter plasmid and phRL-SV40 for 24 h and then the cells were lysed in a passive lysis buffer. A 20 μL aliquot was used for luminescence measurement with a luminometer (Promega), according to the manufacturer's protocol. The data were represented as the ratio of firefly to Renilla luciferase activity (relative luciferase activity).

### Mouse metastatic tumor model

In the nude mouse metastatic tumor model, H-CAFs and HCC cells (1:1 ratio) were co-injected into the spleen of nude mice. After 21 days, liver metastases from HCC cells were observed and the whole liver was dissected and stained by H&E.

### Chromatin immunoprecipitation assay (ChIP)

ChIP was performed using the ChIP assay kit (Upstate Cell Signaling Solutions) according to the manufacturer's instructions. Approximately 4.5×10^7^ HCC cells were used in each treatment. After HCC cells were induced to EMT by IL-6 (25 ng/mL) with or without neutralizing antibody, the cells were crosslinked by treatment with 1% formaldehyde for 10 min at room temperature which was terminated by glycine (final concentration, 0.125 M), and were then harvested and incubated in 600 µL SDS lysis buffer containing protease inhibitors for 10 min on ice. Chromatins were sonicated to yield fragments of ~0.5 kb in length. After sonication, the lysate was centrifuged at 13,000 rpm for 10 min at 4 °C. The supernatant was diluted in ChIP dilution buffer (0.01% SDS, 1% Triton X-100, 1.2 mM EDTA, 167 mM NaCl, 16.7 mM Tris-HCl, pH 8.1, with protease inhibitors). After precleared with protein G-agarose, 5% of the supernatant was saved as input DNA. Two µg rabbit p-STAT3 (Ser727) antibody (CST) or rabbit normal IgG (Sigma) was added to the supernatant, followed by incubation overnight at 4 °C with rotation. After wash, immune complexes were eluted with elution buffer (1% SDS, 0.1 M NaHCO_3_, 200 mM NaCl). Part of captured immune-complex was subjected to western blotting with another human p-STAT3 antibody (BD) to detect whether captured chromatins contained p-STAT3. Crosslinking was reversed by heating at 65 °C overnight. RNA was degraded with RNAase A for 30 min and protein was degraded with proteinase K for 2 h. DNA was purified using the EZChIP polypropylene spin column and subjected to polymerase chain reaction (PCR) amplification using the primers spanning the banding site on TG2 promoter (forward, 5'-CCAGATCTGACCTAA-3'; reverse, 5'-GTCTGAGTCTGTGGGT-3').

### Statistical analysis

Statistical analyses were performed using the SPSS 20.0 software (SPSS, USA). The Students *t*-test was used for comparison of continuous variables between groups. The χ^2^ test was performed and the multivariable logistic regression models were applied to analyze the correlations of the density of H-CAFs with clinicopathologic parameters. The Kaplan-Meier method together with the log-rank test was used for survival analysis. Multivariable Cox regression models adjusting for sex, age (<50 *vs.* ≥50 y), HBV infection, liver cirrhosis, α-fetoprotein (AFP) level (<400 *vs.* ≥400 μg/L), tumor TNM stage (I-II *vs*. III-IV), size (<5 *vs*. ≥5 cm), differentiation grade (well *vs*. moderate-poor), and vascular invasion were further computed to assess independent associations of the density H-CAFs with survival, and the proportional hazard assumption was verified for all variables before conducting further analyses. A two-tailed *P* <0.05 indicated statistical significance.

## Conclusion

In this study, we screened the soluble factors secreted by CAFs and preliminarily confirmed that IL-6, but not HGF or others contributed to EMT of HCC cells. We provided evidence showing that the activation of the IL-6/IL6R/STAT3 axis was essential for H-CAFs-induced EMT by increasing TG2 expression in HCC cells.

## Supplementary Material

Supplementary figures.

## Figures and Tables

**Table 1:** The density of H-CAFs related to clinical features of HCC patients

	Density of H-CAFs		*P* value
	Low (n=34)	Moderate & High (n=90)	
**Tumor size**			
**<**5cm	23	46	0.098
≥5cm	11	44	
**Tumor multiplicity**			
Single	27	65	0.414
Multiple	7	25	
**HBV infection**			
Negative	6	4	**0.025**
Positive	28	86	
**Liver cirrhosis**			
Negative	10	10	**0.013**
Positive	24	80	
**Histological grade**			
Poor and Moderate	25	82	**0.011**
Well	9	8	
**Vascular invasion**			
Negative	29	71	0.421
Positive	5	19	
**TNM stage**			
I+II	22	41	0.057
III+IV	12	49	
**Serum AFP**			
<400 ug/L	22	46	0.175
≥400 ug/L	12	44	

**Table 2:** Multivariate Cox proportional-hazards analysis in the overall HCC patients

Variable	PFS			OS		
	HR	95% CI	*P* value	HR	95% CI	*P* value
Age (<50 y *vs*. ≥50 y)	0.995	0.628-1.578	0.985	0.914	0.531-1.573	0.746
Gender (Male *vs*. Female)	2.254	0.648-7.835	0.201	1.905	0.405-8.965	0.415
Tumor size (<5cm *vs*. ≥5cm)	2.689	1.325-5.460	0.006	2.783	1.284-6.034	0.010
Tumor multiplicity (Single *vs*. Multiple)	1.430	0.833-2.455	0.195	1.726	0.944-3.157	0.076
HBV infection (Negative *vs*. Positive)	1.538	0.500-4.728	0.452	0.904	0.231-3.542	0.884
Liver cirrhosis (Negative *vs*. Positive)	0.894	0.455-1.756	0.745	1.286	0.526-3.142	0.582
Histological grade (Poor and Moderate *vs*. Well)	0.733	0.337-1.593	0.433	0.474	0.164-1.371	0.168
Vascular invasion (Negative *vs*. Positive)	1.438	0.783-2.640	0.242	1.623	0.831-3.169	0.156
TNM stage (Ⅰ+Ⅱ *vs*. Ⅲ+Ⅳ)	0.575	0.262-1.263	0.168	0.795	0.324-1.956	0.618
Serum AFP (<400ug/L *vs*. ≥400 μg/L)	1.348	0.856-2.124	0.198	1.025	0.595-1.768	0.928
Density of H-CAFs (Low *vs*. Moderate and High)	2.348	1.259-4.380	0.007	2.318	1.027-5.232	0.043

**Table 3:** Summary of protein spot identified by MALDI-TOF/TOF MS. Spot numbers refer to those spots in Figure 4

Spot No.	Protein name	Ac.	Score	*P* value	Theoretical		Changes	
					MW (kDa)	pI	Av. Ratio	T-test
**Up-regulated proteins**								
332	Cytokeratin-10	K1C10_HUMAN	96	5.60E-06	59.0	5.13	1.52	0.014
896	Protein-glutamine gamma-glutamyltransferase 2	TGM2_HUMAN	425	6.4E-39	78.4	5.11	4.27	7.10E-06
916	Ezrin	EZRI_HUMAN	123	1.00E-08	69.5	5.94	1.59	0.0098
990	Moesin	MOES_HUMAN	523	1.00E-48	68.0	6.08	1.88	0.0026
1306	Prolyl 4-hydroxylase subunit alpha-1	P4HA1_HUMAN	132	1.30E-09	61.3	5.7	1.7	0.034
1517	Endoplasmin	ENPL_HUMAN	185	6.40E-15	92.696	4.76	1.86	0.0054
1721	Calumenin	CALU_HUMAN	194	8.10E-16	37.198	4.47	1.67	0.014
1727	Gamma-enolase	ENOG_HUMAN	335	6.40E-30	47.581	4.91	2.35	8.20E-05
1881	Leukocyte elastase inhibitor	ILEU_HUMAN	305	6.40E-27	42.829	5.9	1.65	0.0012
1923	Keratin, type I cytoskeletal 19	K1C19_HUMAN	525	6.40E-49	44.079	5.04	1.51	0.0084
2357	Annexin A5	ANXA5_HUMAN	353	1.00E-31	35.971	4.94	1.59	0.0046
2602	Peroxiredoxin-4	PRDX4_HUMAN	167	4.10E-13	30.749	5.86	1.65	0.017
**Down-regulated proteins**								
583	Heat shock 70 kDa protein 4	HSP74_HUMAN	443	1.00E-40	95.127	5.11	-1.59	0.004
1238	Prelamin-A/C	LMNA_HUMAN	68	0.0032	74.38	6.57	-1.74	0.049
1984	Keratin, type II cytoskeletal 1	K2C1_HUMAN	65	0.0066	66.17	8.15	-1.8	0.047
2372	Delta(3,5)-Delta(2,4)-dienoyl-CoA isomerase, mitochondrial	ECH1_HUMAN	208	3.20E-17	36.136	8.16	-1.87	0.084

## Abbreviations

CAFs: Cancer-associated fibroblasts
HCC: hepatocellular carcinoma
H-CAFs: hepatocellular carcinoma-derived CAFs
EMT: epithelial-mesenchymal transition
TG2: transglutaminase 2
IL-6: interleukin-6 (IL-6)
HGF: hepatocyte growth factor
STAT3: signal transducer and activator of transcription 3
OS: overall survival
PFS: progression-free survival
CM: conditional medium
NSF: normal skin fibroblast
IL6R: IL-6 receptor
2-D DIGE: two-dimensional difference in gel electrophoresis
MALDI-TOF/TOF MS: matrix-assisted laser desorption/ionization-time of flight (TOF)/TOF mass spectrometry
