# Supplementary material for: Cancer-associated Fibroblasts induce epithelial-mesenchymal transition via the Transglutaminase 2-dependent IL-6/IL6R/STAT3 axis in Hepatocellular Carcinoma
Source: Int J Biol Sci. 2020 Jul 19;16(14):2542–58. doi: 10.7150/ijbs.45446 (PMC7415430; doi:10.7150/ijbs.45446)
Supplement: Supplementary file 1 — Supplementary figures. [file ijbsv16p2542s1.pdf]

Figure S1

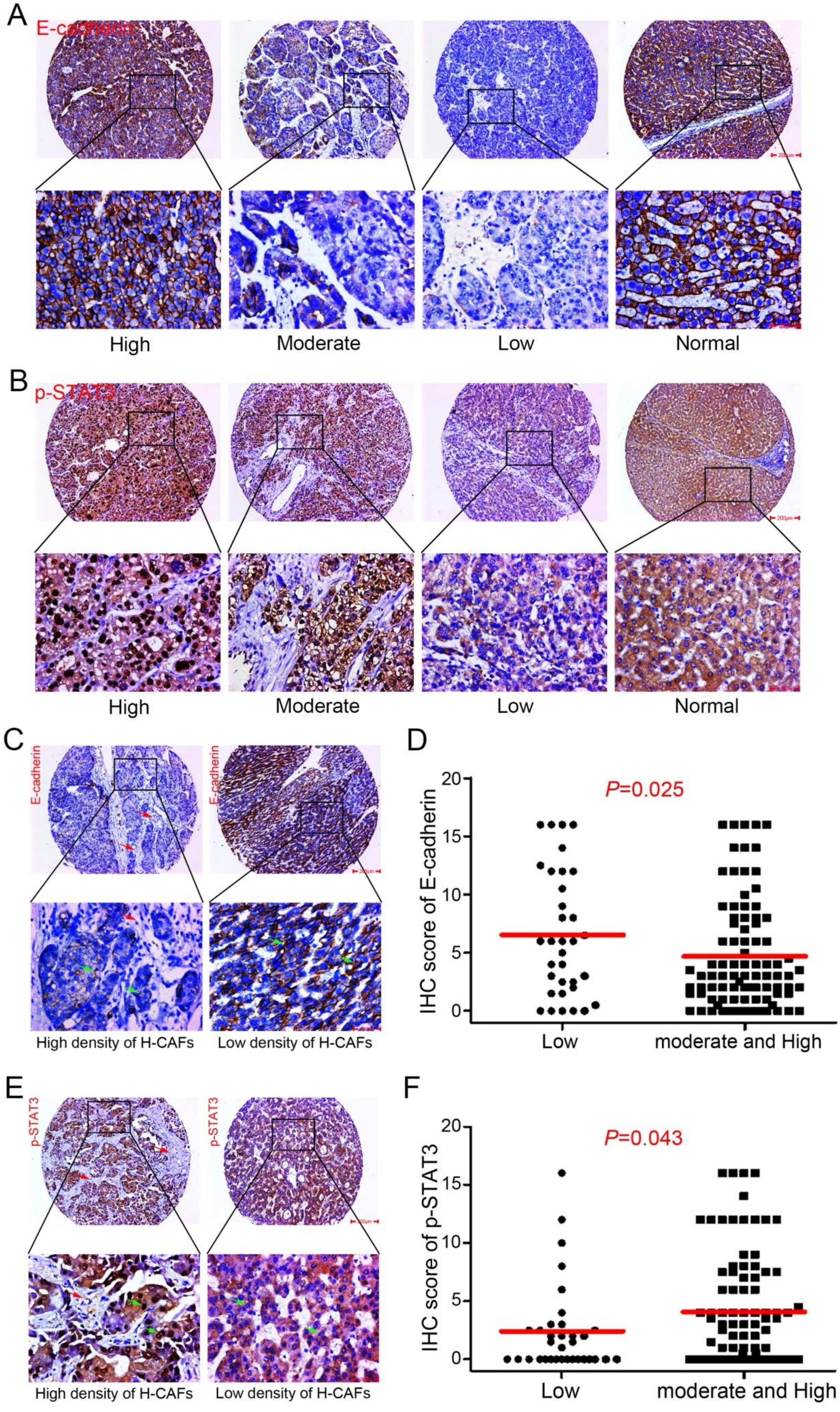
A
E-cadherin
High
Moderate
Low
Normal
B
p-STAT3
High
Moderate
Low
Normal
C
E-cadherin
E-cadherin
High density of H-CAFs
Low density of H-CAFs
D
P=0.025
IHC score of E-cadherin
20
15
10
5
0
Low
moderate and High
E
p-STAT3
p-STAT3
High density of H-CAFs
Low density of H-CAFs
F
P=0.043
IHC score of p-STAT3
20
15
10
5
0
Low
moderate and High

Figure S1. Immunohistochemistry (IHC) analysis of H-CAF density and the expressions of E-cadherin and p-STAT3 in HCC patients. (A) E-cadherin was lowly or moderately expressed in the cancer cell cytoplasm, whereas it was highly expressed in well-differentiated or normal tissue (original magnification, 100×). An enlarged view is shown in the lower panel (original magnification, 400×). (B) p-STAT3 was highly or moderately expressed in the cancer cell nucleus, whereas it was lowly expressed in well differentiated or normal tissue (original magnification, 100×). An enlarged view is displayed in the lower panel (original magnification, 400×). (C) A high density of H-CAFs in the samples was associated with low E-cadherin expression, whereas a low density of H-CAFs was associated with high E-cadherin expression (original magnification, 100×). The lower panel shows the enlarged view (original magnification, 400×). (D) IHC scores of E-cadherin were compared in the subgroups with dichotomized H-CAFs density (low VS moderate and high). (E) Correlation of H-CAFs density with p-STAT3 expression. (The p-STAT3 and E-cadherin expression, brown color, green row; H-CAFs, unstained, red row). (F) IHC scores of p-STAT3 were compared in the subgroups of H-CAFs density.

Figure S2

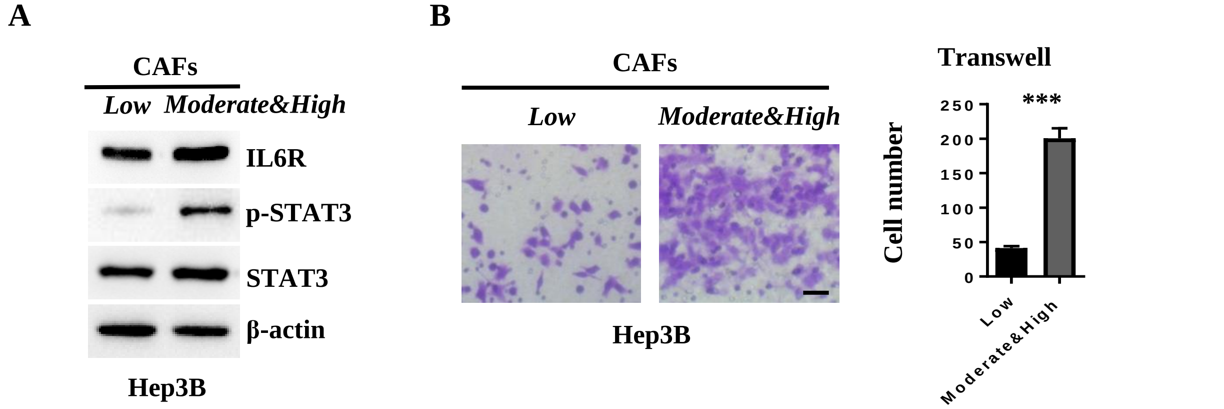

Figure S2. (A) Western blotting and (B) transwell analysis of Hep3B, treated with H-CAFs*low* or H-CAFs*moderate/high*, were performed.

Figure S3

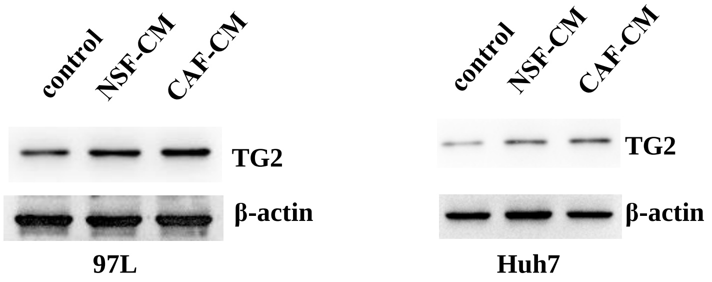

Figure S3. TG2 expression was tested in MHCC-97L and Huh7 cell lines by western blotting.

Figure S4

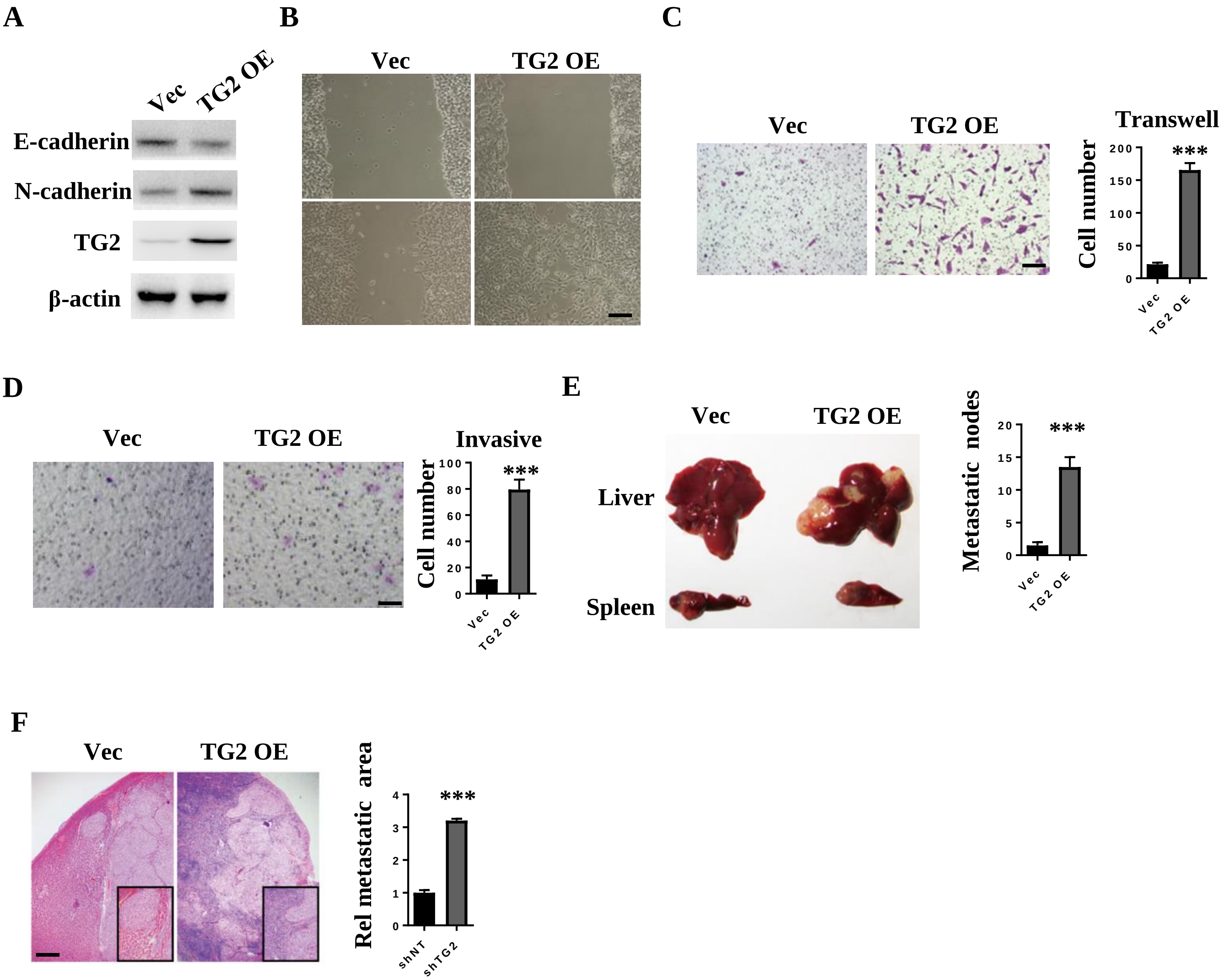

Figure S4. Overexpression of TG2 induced EMT of HCC cells. The error bars represent ± SEM; * $P<0.05$, *** $P<0.001$). (A) TG2 was stably overexpressed by CMV promoter in Huh7 cells. An empty vector was used as a control. N-cadherin and E-cadherin were used as indicators of EMT initiation. (B) Wound healing, (C) transwell, and (D) invasive assays were performed using Huh7-Vec and TG2 OE cells. (E) In the nude mouse metastatic tumor model, Huh7-Vec or TG2 OE cells were injected into the spleen of nude mice. After 21 days, the tumor of liver metastases was counted and then confirmed by H&E staining (F). Original magnification, 50×.
